# Profile of access and use of medicines in the Brazilian population – contributions and challenges of PNAUM – Household Survey

**DOI:** 10.1590/S1518-8787.201605000SUPL2AP

**Published:** 2016-12-01

**Authors:** Jorge Antonio Zepeda Bermudez, Marilisa Berti de Azevedo Barros

**Affiliations:** I Fundação Oswaldo Cruz. Rio de Janeiro, RJ, Brasil; II Departamento de Saúde Coletiva. Faculdade de Ciências Médicas. Universidade Estadual de Campinas. Campinas, SP, Brasil

With the scientific and technological advances achieved in the last decades in the health field, in particular those related to diagnoses and treatments, the relevance and essentiality of the use of medicines for the treatment, control, and prevention of diseases tended to grow. In a world where the capitalistic organization prevails in the production and distribution of health products, and where the human needs do not fundamentally govern these processes of production and consumption of goods and services, many distortions and irrationalities appear in the patterns of drug use and access, as well as in health services in general.

It is no coincidence that the issue of access to medicines has been object of discussions in various forums of Global Health. Recent episodes, such as the launch of new antiviral medicines to treat hepatitis C or new oncologic products, all at prices considered inaccessible even to the health systems of high-income countries, straighten the problem of relative prices and costs of the current system of research and development, having the intellectual property as a barrier to access^3^. The subject raised such interest that led the Secretary-General of the United Nations to establish a high-level panel to discuss the problem, making it clear that this issue could no longer be restricted only to the World Health Organization, but extrapolated for political discussion at the highest level^16^. Additionally, the issue of access to medicines today is no longer restricted as a problem of poor countries or populations, but is a matter of global importance.

In this context, it is fundamental to define social policies by the needs of the population, especially the most vulnerable and socially disadvantaged segments, and to bring fairness and rationality in the access to health care and treatment. The project of conducting a national survey of medicines is part of the perspective of evaluation and monitoring of performance against the goals that guided the policies of health and pharmaceutical assistance developed in Brazil. The Pesquisa Nacional sobre *Acesso, Utilização e Promoção do Uso Racional de Medicamentos no Brasil (*PNAUM – National Survey on Access, Use and Promotion of Rational Use of Medicines in Brazil) constitutes a significant milestone in the composition of nationwide surveys that have been carried out in Brazil, as well as a historical record that allows the monitoring of the effect of changes caused by the economic austerity policies that lie on the horizon.

The studies presented and discussed in this thematic issue of the RSP, based on the PNAUM (Household Survey Section), a population-based cross-sectional study that collected data between September 2013 and February 2014, undoubtedly, contributes decisively to the best knowledge of the public policy of the pharmaceutical sector in Brazil, the changes promoted in the public and private demands and, above all, the initiatives that aim to increase the access of our populations to essential medicines.

Gadelha et al.^8^, when contextualizing the construction process of the PNAUM, also feature elements of public policy related to Science, Technology, and Innovation in Health that had been developed and implemented in Brazil at the time when this national survey was conducted. Opening the collection of articles, generated in the context of this research, the authors present conceptual aspects and prospects of development and consolidation of strategies for pharmaceutical policies by the Ministry of Health.

Analyzing a set of relevant topics in the context of appropriate pharmaceutical care and rational use of medicines, the articles in this supplement provide the opportunity to enjoy various aspects and results of pharmaceutical care policies, such as the expansion of the list of medicines available in the public health service network, the Popular Pharmacy Program, and the provision of free medication to specific diseases.

Among the articles presented, and to allow better contextualization of the methodology, Mengue et al.^10^ describe in detail the methodological aspects that guided the household survey of PNAUM, including the design of the study, the sampling process, the instruments for data collection, and the implementation in the field. Producing a sample that allows inferences to the urban areas and macro-regions of the Country, the survey collected data from 41,433 individuals in more than 20,000 households, being the first major national survey on access and use of medicines. The authors highlight the relevance of their results as a baseline for future evaluation studies on the impacts of government policies and actions in the fields of access and use of medicines, allowing the monitoring of the trend of these events among regions and social groups. The future utility of this research is another element valuable to consider.

Some of the articles of this supplement focus on the characterization of the general profile of drug use, whether for the whole of the population or for specific demographic segments. Bertoldi et al.^4^ analyze the global prevalence of medicine, showing the high percentage of Brazilians (50.7%) that use medicines and confirming that medicines for acute conditions are more consumed (33.7%) in comparison with the ones intended for chronic diseases (24.3%). They also noted that the usage patterns differ among age groups and regions of the Country.

The article focuses on the use of medicines by children under 12 years of age, da Silva Dal Pizzol et al.^6^ reviewed the data of 7,528 children. The authors noted that 31% of them used some medication and that most of it was used for acute health problems (27.1%), and 5.6% for chronic diseases. The study identified the significant regional variability and the types of medicines most often used by children.

Other age group that deserved particular analysis in this supplement was that of the older adults, which constitute, in a scenario of accelerated population aging and high prevalence of chronic diseases, the segment with the highest rates of medicine consumption. Considering only the medicines used for eight chronic diseases surveyed by the PNAUM, the study by Ramos et al.^13^ on polypharmacy in older adults identified that 18% of older people use at least five medications. The researchers also analyzed the diversity of this percentage among regions, among individuals who own or not private health plan and according to the presence of obesity.

Given the relevance of the chronic diseases in the Brazilian morbidity and mortality profile, the supplement includes three articles that address features of medicine consumption intended for all of these diseases or specifically for one of them, hypertension. Oliveira et al.^12^, evaluating the access to medicines for the treatment of chronic non-communicable diseases, they observed that 94.3% of all Brazilians with one of the chronic diseases researched by PNAUM, who had medication prescription for the control of the disease, had access and used the medication prescribed. It should be stressed that such access was total, i.e., to all the medication prescribed. Only 2.6% of individuals had not taken the medicines they needed in the past 30 days. The authors believe that national policies of medicines and pharmaceutical assistance implemented in Brazil since 1999 is reaching the goal of expanding access and reducing inequalities.

Tavares et al.^14^ analyzed the free access to medicines for chronic diseases and reported that 47.5% of Brazilians with these diseases obtained all the medicines they needed free of charge. The percentage of free access was higher among individuals of low socioeconomic status, but with differences regarding geographic regions and classes of medicines. The authors indicate the reduction of socioeconomic inequalities of access with the provision of free medicines.

Studying the access to medicines for hypertension, Mengue et al. identified that of all the hypertensive people who knew their condition and who had received a prescription for treatment, 94.6% were using medication, being the total access obtained by 97.9% of them. The authors relate the number of medicines used by hypertension people and the types of medication most used by them. The article indicates that 72.0% obtained the medicines in the Brazilian Unified Health System (SUS) or by the Popular Pharmacy Program, and that only 25.7% of hypertensive Brazilians paid for the medication.

Research on catastrophic health expenditure have been developed around the world and the study of this supplement that focused on this theme, conducted by Luiza et al.^9^, detected that catastrophic health expenditure occurred in 5.3% of Brazilian households and medicines were one of the items mentioned in 3.2%. Catastrophic expenditure was considered when the individual stopped buying something to pay for health expenses. The percentage of catastrophic household expenditure proved to be inferior to those of other countries, as verified in prior studies developed in various countries and in Brazil^2,17^. The authors^9^ conclude that Brazilian policies appear to be protecting the families from these expenses.

The provision of generic medication was one of the policies developed in the Country to increase the access of the population to medicines. Bertoldi et al.^4^, in their study, detected that 45.5% of the medicines used by the Brazilian population are generic, as well as 37.3% of those provided by SUS. The authors analyze the prevalence of the use of generics, stratifying by economic classes and other variables including sex, age, schooling, and region of the Country.

To evaluate the rational use of medicines, the practice of self-medication and adherence of patients to the prescriptions were also contemplated in this supplement. Self-medication was the object of study of Arrais et al.^1^, who found a higher prevalence of this practice in the Northeast region and in the female population, with painkillers and muscle relaxers being the predominant categories. The findings confirm that self-medication is common practice in Brazil and the authors warn of the possible risks associated with it.

Tavares et al.^14^, evaluating the factors associated with low adhesion to the treatment of chronic diseases, found 30.8% of low adhesion and verified that the problem is more prevalent in the Northeast and Midwest regions of the Country. The authors indicate the actions required to improve the levels of adhesion.

Another topic covered in this supplement, considering the high frequency of use and relevance in sexual and reproductive health policies, was the access to contraceptives. Farias et al.^7^, addressing the use and access to oral and injectable contraceptives, reported that 28.2% of Brazilian women between 15 and 49 years use oral and 4.5% use injectable contraception. Most of them paid for the contraceptive, buying them in commercial pharmacies.

All the articles published in this supplement provides a comprehensive overview of multiple significant aspects on access and use of medicines in the context lived by the Brazilian population. The results show the importance of maintenance and expansion of health and pharmaceutical policies directed to the promotion of equity in health care and access to the rational use of medicines.
